# Generation of West Nile Virus Infectious Clones Containing Amino Acid Insertions Between Capsid and Capsid Anchor

**DOI:** 10.3390/v6041637

**Published:** 2014-04-09

**Authors:** Rianna Vandergaast, Lisa I. Hoover, Kang Zheng, Brenda L. Fredericksen

**Affiliations:** Maryland Pathogen Research Institute and Department of Cell Biology and Molecular Genetics, University of Maryland, College Park, Prince George’s County, MD 20742, USA; E-Mails: rvanderg@umd.edu (R.V.); linjaian@umd.edu (L.I.H.); kzheng@umd.edu (K.Z.)

**Keywords:** West Nile virus, WNV, infectious clone, reporter, virus replication

## Abstract

West Nile virus (WNV) is a positive-sense RNA arbovirus responsible for recent outbreaks of severe neurological disease within the US and Europe. Large-scale analyses of antiviral compounds that inhibit virus replication have been limited due to the lack of an adequate WN reporter virus. Previous attempts to insert a reporter into the 3’ untranslated region of WNV generated unstable viruses, suggesting that this region does not accommodate additional nucleotides. Here, we engineered two WNV infectious clones containing insertions at the Capsid (C)/Capsid Anchor (CA) junction of the viral polyprotein. Recombinant viruses containing a TAT(1-67) or Gaussia Luciferase (GLuc) gene at this location were successfully recovered. However, rapid loss of most, if not all, of the reporter sequence occurred for both viruses, indicating that the reporter viruses were not stable. While the GLuc viruses predominantly reverted back to wild-type WNV length, the TAT viruses retained up to 75 additional nucleotides of the reporter sequence. These additional nucleotides were stable over at least five passages and did not significantly alter WNV fitness. Thus, the C/CA junction of WNV can tolerate additional nucleotides, though insertions are subject to certain constraints.

## 1. Introduction

**Figure 1:**
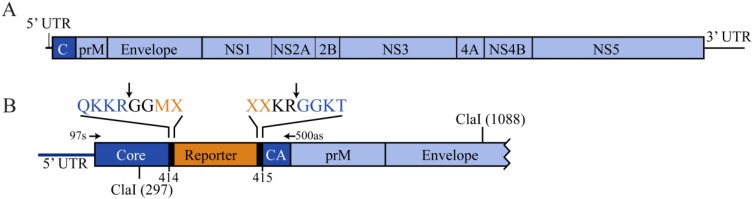
Schematic of West Nile virus-New York (WNV-NY) reporter viruses. (**A**) WNV genome. A scale representation of the WNV genome is shown. The arrangement of the 5’ and 3’ untranslated regions and the ten genes are indicated; (**B**) WNV-NY reporter viruses. Reporter genes (orange) were inserted between nucleotides (nts) 414 and 415 (Capsid (C)/Capsid Anchor (CA) junction) of WNV-NY (blue). Heavy black bars represent additional nts that form the engineered WNV-NS2B/NS3 protease cleavage sites; the P_4_-P_4_’ residues and site of protease cleavage (↓) are indicated at the top, where X represents variable amino acids. The location of the two ClaI sites used in the generation of the reporter-containing pA constructs is also shown, as are the annealing sites and orientations of the WNV-500as and WNV-97s primers used for RT-PCR analyses.

West Nile virus (WNV) is an ~11 kb, enveloped, positive-sense RNA virus belonging to the family *Flaviviridae*. The viral genome consists of a single open reading frame flanked by 5’ and 3’ untranslated regions (UTRs) (Figure 1A). The incoming viral RNA is translated by the host cell to generate a single polyprotein, which is co- and post-translationally cleaved by viral and cellular proteases, liberating three structural proteins (capsid, pre-membrane, and envelope) and seven nonstructural proteins (NS1, NS2A, NS2B, NS3, NS4A, NS4B, and NS5). Historically, WNV infections have been associated with mild febrile illness. However, recent outbreaks within the Americas, Europe, and Israel have been marked by a dramatic increase in the incidence of severe neurological diseases, including meningitis, encephalitis, and acute flaccid paralysis [1,2,3,4,5]. Since 1999, there have been over 17,000 cases of WNV with neurological complications, and over 1600 deaths due to WNV in the United States [6], making WNV the leading cause of mosquito-borne neuroinvasive disease. Currently, treatment for WNV infections is limited to supportive care. Therefore, rapid and high-throughput methods for identifying antiviral agents are necessary to expedite the development of treatment options for WNV. 

Evaluation of the efficacy of antiviral agents has traditionally relied on monitoring the effect of compounds on either viral yields, using standard plaque assays, or viral genome levels, using quantitative reverse transcription (RT)-PCR. However, these assays are time consuming, limiting their usefulness for large-scale screens of compound libraries. Therefore, multiple groups have developed subgenomic replicon systems for WNV that encode reporter genes, which serve as an easily detectible surrogate marker for viral replication [7,8,9,10,11,12,13]. Several of these reporter-expressing replicon systems have been used in high-throughput screens (HTSs) to identify small-molecule inhibitors of WNV replication [10,11,12,13]. While replicons do not require higher-level containment facilities, they also do not permit complete and authentic evaluation of the antiviral effects of compounds on the viral life cycle. Because the replicon system can only assess the effect of potential antiviral compounds on viral genome replication or steps prior to viral genome replication, HTSs utilizing replicons may miss compounds that inhibit other steps in the virus life cycle. 

Due to the limitations of the replicon systems, the development of an authentic, full-length reporter virus is needed. Initial attempts to construct a full-length reporter virus incorporated the gene for either Green Fluorescent Protein (*gfp*) or *Renilla* luciferase into the WNV 3’ UTR [14,15]. Although infectious virus was recovered from both constructs, the viruses were unstable, and expression of the reporter proteins dropped precipitously after several passages. Moreover, the rate of replication of the GFP-containing virus was reduced compared to wild-type WNV [14]. Insertion of the *gfp* gene into the 3’ UTR of a WNV replicon system also decreased replication efficiency [16], suggesting that WNV does not tolerate nucleotide insertions within the 3’ UTR. In a separate attempt to generate a stable reporter virus, the *gfp* gene, followed by 51 nucleotides (nts) of foot-and-mouth disease virus (FMDV) protease 2A, were incorporated into a duplicate copy of the viral capsid gene located at the 5’ end of the WNV coding region [17]. The recovered virus was stable, but exhibited delayed replication kinetics and decreased fitness compared to wild-type WNV. 

Here, we set out to generate a stable WN reporter virus with replication kinetics similar to wild-type virus. We synthesized two WNV infectious clones containing either a TAT(1-67) or Gaussia luciferase (GLuc) reporter at the Capsid (C)/Capsid Anchor (CA) junction (Figure 1B). Evidence suggests that this location may tolerate additional nucleotides [18]. Schrauf *et al.* replaced the WNV C/CA protease cleavage site with 20 residues of the FMDV protein 2A, which contained a Pro-Gly-Pro motif that directed immediate separation of C from CA during translation. This recombinant virus, WNV-2A, replicated well in mosquito cells but exhibited a small-plaque phenotype in Vero cells. The appearance of compensatory mutations in CA that restored a large plaque phenotype, suggest that the nature, and not the location, of the insert may account for the instability and reduced fitness of this virus in Vero cells. Here, we successfully recovered recombinant viruses encoding the full-length TAT(1-67) and GLuc reporters, though the recovered viruses were unstable and quickly lost large portions of the reporter genes. Nevertheless, our findings indicated that WNV tolerates at least 75 additional coding nucleotides at this site without negatively impacting viral fitness. The C/CA junction, therefore, represents a potential site for manipulation to generate recombinant WNVs. 

## 2. Results and Discussion

### 2.1. Generation of WNV-NY/TAT

To overcome the limitations of instability and reduced viral fitness associated with WN reporter viruses, we sought to generate a recombinant virus in which the reporter was located within the coding region of the viral genome and liberated from the polyprotein via cleavage by the viral protease. Since insertion of *gfp* at the 5’ end of the viral coding region is stable [17], we focused on this region. The WNV NS2B-NS3 protease naturally cleaves the viral polyprotein between Capsid (C) and Capsid Anchor (CA) [19,20,21], making this position an attractive site for insertion of a reporter gene. Because larger inserts are potentially detrimental to viral fitness, we minimized the size of the sequence inserted at this location. To this end, we began by inserting the sequence encoding residues 1-67 of HIV-TAT, which is the minimal portion of TAT required to drive efficient expression from the HIV long-terminal repeat (LTR) [22]. Although the HIV-TAT protein is not itself a reporter, several cell lines have been developed that express TAT-responsive reporters. For HTSs with WNV, similar TAT-responsive reporters can be stably transfected into cell lines of interest. Thus, TAT(1-67) provided us with the opportunity to probe the flexibility of the WNV C/CA junction using an insert sequence substantially smaller than currently available reporters. In order to ensure that the TAT(1-67) sequence was proteolytically separated from the rest of the WNV polyprotein, we engineered two Gly residues and a Lys-Arg residue at the N- and C-terminus, respectively, of the TAT(1-67) insert (Figure 1B). Addition of these amino acids preserved the wild-type WNV C/CA cleavage site (P_2_-P_2_’) at both ends of TAT(1-67) and did not affect the ability of the TAT protein to stimulate HIV LTR-driven expression (Figure S1). There is substantial natural variation between the P3-P6 positions of the WNV protease, and evidence suggests that substrate specificity is almost exclusively governed by the P2-P2’ residues [23,24,25].

**Figure 2:**
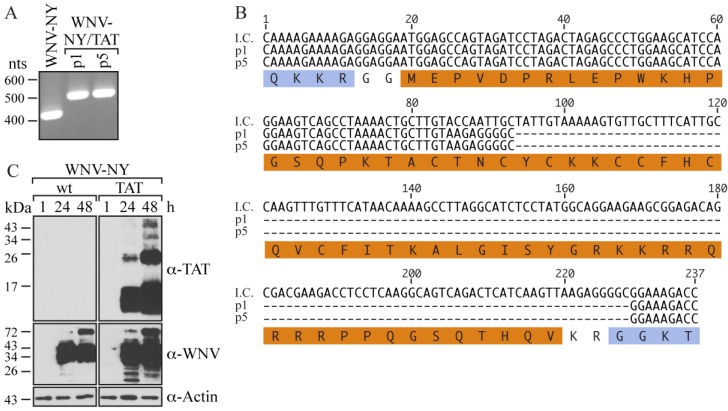
Characterization of WNV-NY/TAT. (**A**) RT-PCR analysis. RNA extracted from passage 1 (p1) and passage 5 (p5) WNV-NY/TAT-infected cells at 48 h post-infection was used as a template for RT-PCR amplification of the WNV C/CA region (WNV nts 97-500). RNA extracted from cultures infected with wild-type WNV-NY was used as a positive control. DNA molecular size standards (in nts) are indicated on the left; (**B**) Sequences. The p1 and p5 PCR products (Panel A) were sequenced by GeneWiz. The sequence encompassing the TAT(1-67) insertion in the WNV-NY/TAT infectious clone (I.C.) is shown at the top. The amino acid sequence is shown below with the WNV residues indicated in blue and the TAT(1-67) residues in orange; (**C**) Immunoblots. Vero monolayers were infected (MOI = 0.05) with WNV-NY or WNV-NY/TAT p0. Cell lysates prepared at the indicated times (h) were subjected to SDS-PAGE and immunoblot analysis with anti-TAT (top), anti-WNV (middle), or anti-actin (bottom) antibodies. Protein molecular size standards (in kilodaltons) are indicated on the left.

To create the reporter virus, we inserted the modified TAT sequence into a highly virulent strain of WNV, WNV-New York (WNV-NY), which is of interest for high throughput studies. Vero cells were electroporated with *in vitro-*transcribed *WNV-NY/TAT* RNA. Although no cytopathic effects (CPE) were observed, a relatively high titer viral stock (p0) (2.5 × 10^6^ plaque-forming units (pfu)/mL), with a plaque phenotype indistinguishable from that of wild-type WNV-NY [26], was recovered at 7 days post-electroporation. Passage of this viral stock in Vero cells generated a 4.9 × 10^7^ pfu/mL (p1) stock. To assess the integrity of the *TAT(1-67)* gene within the recovered virus, total RNA was extracted from p1 cultures and used as a template for reverse transcription (RT)-PCR with primers spanning the site of insertion. As expected, amplification of wild-type WNV-NY RNA resulted in the production of a ~400 nts band (Figure 2A). In contrast, the PCR product generated from the amplification of WNV-NY/TAT p1 RNA was 500 nts, which despite being larger than that of wild-type WNV-NY, was approximately 100 nts smaller than expected for a virus containing TAT(1-67). Based on this size difference, we concluded that the recovered virus contained only part of TAT(1-67). Indeed, sequencing indicated that the recovered virus contained only the first 25 residues of TAT (Figure 2B), suggesting that the TAT(1-67) sequence was unstable in this context. Not surprisingly, we were unable to detect functional TAT protein using the TZM-bl reporter cell line (Figure S2), which is consistent with TAT’s requirement of residues 1-67 for efficient trans-activation of the HIV LTR [22].

To evaluate whether the truncated TAT sequence was stable within the WNV-NY genome, we serially passaged the p1 virus in Vero cells four more times. The RT-PCR product generated from passage 5 (p5) RNA was the same size as that generated from p1 RNA (Figure 2A). Moreover, the sequences of the p1 and p5 RT-PCR products were identical, indicating that the truncated TAT(1-25) sequence was stable within the WNV-NY genome. Thus, WNV can tolerate the addition of coding nucleotides at the C/CA junction, though certain size or sequence restrictions exist. 

To assess whether the TAT(1-25) insert was proteolytically separated from the rest of the WNV-NY/TAT polyprotein, we examined TAT expression in Vero cells infected with WNV-NY/TAT (p0). Although TAT-reactive proteins were detected in cultures infected with WNV-NY/TAT p0, most bands were larger than 2.5 kilodaltons (kDa), the size expected for TAT(1-25) (Figure 2C). Thus, TAT(1-25) was not completely cleaved from the WNV-NY/TAT polyprotein.

**Figure 3:**
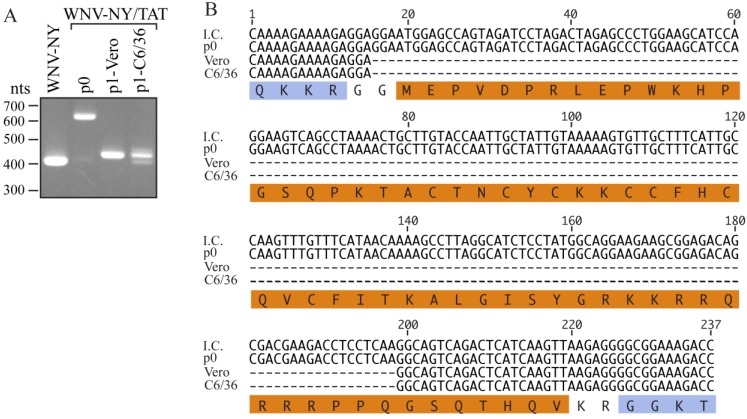
Passage of WNV-NY/TAT on insect cells. (**A**) RT-PCR analysis. RNA was extracted from cells transfected with *WNV-NY/TAT* RNA (p0) and Vero or C6/36 monolayers infected with the p0 virus (p1). The extracted RNA was used as template for RT-PCR amplification of the WNV C/CA region (WNV nts 97-500). RNA recovered from cultures infected with wild-type WNV-NY was used as a positive control. DNA molecular size standards (in nts) are indicated on the left; (**B**) Sequences. The p0 and p1 PCR products (Panel A) were sequenced by GeneWiz. The sequence encompassing the TAT(1-67) insertion in the WNV-NY/TAT infectious clone (I.C.) is shown at the top. The amino acid sequence is shown below with the WNV residues indicated in blue and the TAT(1-67) residues in orange.

To determine whether portions of the TAT(1-67) sequence were lost during the initial recovery of the virus or during the subsequent (p1) passage, we performed a second recovery of WNV-NY/TAT from Vero cells electroporated with *WNV-TAT(1-67)* RNA. Similar to the first recovery, CPE was not observed within the electroporated monolayer. However, the titer of the stock recovered at 7 days post-transfection was substantially lower (35 pfu/mL) than in the initial recovery. Moreover, RT-PCR of total RNA isolated from the electroporated monolayer (p0) yielded a 600 nts band (Figure 3A), consistent with the presence of the TAT(1-67) insert. Sequencing confirmed the presence of the entire TAT(1-67) coding region as well as coding sequence for the WNV protease cleavage sites (Figure 3B). Therefore, insertion of the entire TAT cassette did not abrogate WNV-NY infectivity. However, as previously observed, a large portion of the TAT insert was deleted during the first passage of this virus. 

Since extension of the WNV Capsid protein negatively affects WNV replication in Vero cells, but not mosquito cells [18], we hypothesized that the TAT(1-67) sequence may be more stable in insect cells. Therefore, the recovered WNV-NY/TAT p0 virus was passaged once in Vero or mosquito C6/36 cells. RT-PCR analysis of RNA extracted from the p1 cultures indicated that a substantial portion of the TAT cassette was missing (Figure 3A). Sequence analyses revealed that the WNV-NY/TAT genomes produced during the Vero and C6/36 p1 amplifications retained only the coding sequence for residues 61-67 of the TAT insert (Figure 3B), providing additional evidence that the full TAT(1-67) cassette is not well tolerated at the C/CA junction. It is unlikely that the identical deletions in the TAT(1-67) coding sequence arose independently during passage of the virus in two separate cell lines. Rather, the p0 population likely contained undetectable levels of the truncated WNV-NY/TAT(61-67) in addition to full-length WNV-NY/TAT(1-67). The loss of full-length WNV-NY/TAT(1-67) during passage, also suggested that WNV-TAT(61-67) significantly outcompeted WNV-TAT(1-67) and was preferentially amplified. Therefore, the full-length WNV-NY/TAT virus is not only unstable, but also less fit than wild-type WNV-NY. 

### 2.2. Generation of WNV-NY/GLuc

**Figure 4:**
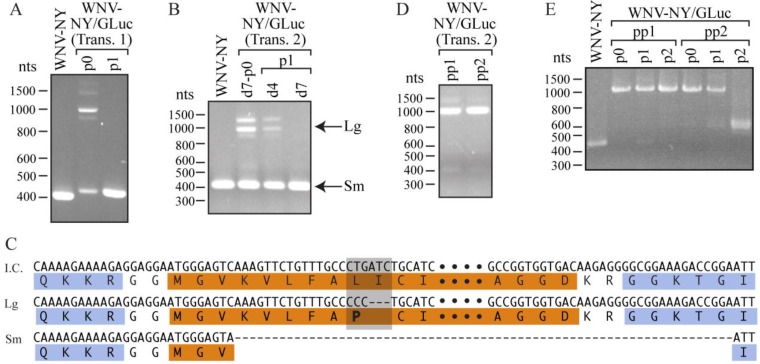
Characterization of WNV-NY/Gluc. (**A**) RT-PCR analysis. RNA extracted from cells transfected with *WNV-NY/TAT* RNA (p0) and cultures infected with the p0 stock (p1) were used as templates for RT-PCR amplification of the WNV C/CA region (WNV nts 97-500). RNA recovered from cultures infected with wild-type WNV-NY was used as a positive control. DNA molecular size standards (in nts) are indicated on the left; (**B**) RT-PCR analysis of passaged WNV-NY/Gluc. Vero cells were transfected with infectious *WNV-NY/GLuc* RNA. A portion of the culture supernatant (p0) was collected after 4 days (d4). The remaining supernatant and total RNA was collected at 7 days (d7) post-transfection. Total RNA was extracted from Vero cells infected with d4 or d7 p0 stocks (d4-p1 and d7-p1). Extracted RNA was used as template for RT-PCR as described in Panel A; (**C**) Sequences. The d7 p0 large (Lg) and small (Sm) PCR products (Panel B) were sequenced by GeneWiz. The sequence encompassing the GLuc-encoding region of the WNV-NY/GLuc infectious clone (I.C.) is shown at the top. The amino acid sequence is shown below with WNV residues indicated in blue and GLuc residues in orange. Bullets represent additional GLuc residues that are not shown but are present in the I.C. and Lg product. The gray box highlights the residues that are different between I.C. and Lg; (**D**) Plaque purification of WNV-NY/Gluc. The d4 p0 stock (Panel B) was also used to inoculate a confluent monolayer of Vero cells, which were then overlaid with 0.9% agarose/DMEM. After 4 days, two plaques (pp1 and pp2) were picked, incubated with 1 mL DMEM, and used to inoculate Vero cells. After an additional 4 days, culture supernatants were collected. RNA was extracted from the infected Vero cells and used as template for RT-PCR as described in Panel A; (**E**) RT-PCR analysis of pp1 and pp2 serial passages. The pp1 and pp2 viruses were passaged twice in Vero cells to generate p1 and p2. RNA extracted from the infected Vero cells was used as template for RT-PCR as described in Panel A.

Our findings suggested that the C/CA junction of WNV can tolerate additional sequences. However, the inability to recover stable full-length WNV-NY/TAT(1-67) suggested that the insert was subject to one or more of the following constraints: (1) the size of the full-length insert exceeded that which is tolerated by WNV; (2) specific sequences within the coding regions for TAT residues 26-60 affected WNV replication; or (3) the cellular activities of TAT(1-67) impeded virus replication. To distinguish between these possibilities, we generated a second infectious clone containing Gaussia luciferase (GLuc) [27,28] instead of TAT(1-67). A viral (p0) stock of 1.65 × 10^4^ pfu/mL was recovered from Vero cells electroporated 7 days earlier with *WNV-NY/GLuc* RNA, though no CPE was evident over this time period. The plaque phenotype of the recovered virus was highly variable. Some plaques were indistinguishable from those of wild-type WNV-NY, while others were significantly smaller [26], suggesting the virus population was mixed. Indeed, when RNA isolated from the infected Vero cells was used as template for RT-PCR, two predominate PCR products were generated (Figure 4A). The size of the smaller product was similar to that of wild-type WNV-NY, and sequence analysis indicated that it had entirely wild-type WNV-NY sequence within the C/CA region. In contrast, as confirmed by sequencing, the larger PCR product contained the entire GLuc insert. To test whether full-length WNV-NY/GLuc was stable within the virus population, we passaged the WNV-NY/GLuc p0 once in Vero cells for 48 h. Using RT-PCR, we readily detected a band of wild-type WNV-NY size, but did not detect any full-length WNV-NY/GLuc (Figure 4A). Thus, wild-type WNV-NY outcompeted WNV-NY/GLuc and had a fitness advantage over WNV-NY/GLuc.

In an attempt to isolate a pure WNV-NY/GLuc population, we repeated the electroporation of Vero cells with *WNV-NY/GLuc* RNA and collected culture supernatant at 4 and 7 days post-transfection. While only 75 pfu/mL of virus was recovered on day 4, the plaques had a uniformly small plaque phenotype [26], indicative of a single virus population. In contrast, virus recovered on day 7 displayed both small- and large-plaque phenotypes, similar to those observed in the previous isolation. RT-PCR analysis of RNA extracted on day 7 post-electroporation produced multiple products of various sizes (Figure 4B). The large, ~1000 nts product contained the full-length GLuc insert, with two nucleotide changes (TG➔CC) at position 26-27 and a three-nucleotide deletion at position 28-30, which together resulted in a two-residue mutation (ALIC➔APC) within the GLuc N-terminus (Figure 4C). The smaller product at ~400 nts was the same size as wild-type WNV-NY, but retained the first 9 nts of the GLuc gene and lacked the first 15 nts of the CA sequence, which led to a KTG➔MGV mutation within the resulting polyprotein. Thus, the day 7 p0 virus was a mixed population. Moreover, the only virus detected by RT-PCR following a single passage of this population in Vero cells was the smaller KTG➔MGV virus (Figure 4B), suggesting that the GLuc sequence, like the TAT(1-67) sequence, was unstable. This instability was further confirmed by the observation that a single passage of the day 4 p0 virus, which had produced only small plaques, yielded a virus stock of mixed small- and large-plaque phenotypes [26] and RT-PCR products of variable lengths (Figure 4B). 

### 2.3. Plaque Purification of WNV-NY/GLuc Virus

To further examine the stability of WNV containing the full-length GLuc insert, we plaque purified two clones from the day 4 p0 stock of WNV-NY/GLuc. The two plaques, pp1 and pp2, were amplified for 96 h in Vero cells (p0). RT-PCR of total RNA isolated from p0 cultures yielded a band of ~1000 nts, suggesting that the population consisted predominately of full-length WNV-NY/GLuc (Figure 4D). Sequence analyses revealed that both pp1 and pp2 had the full-length GLuc(ALIC➔APC) insert. Thus, the ALIC➔APC mutation was introduced prior to 4 days after transfection of the infectious RNA. 

To test the stability of the plaque-purified clones, we passaged the pp1 and pp2 stocks twice in Vero cells and extracted total RNA from the infected cells for RT-PCR analysis and sequencing. While WNV-NY/GLuc(ALIC➔APC)-pp1 retained the full-length GLuc(ALIC➔APC) insert through both passages, WNV-NY/GLuc(ALIC➔APC)-pp2 became a mixed population within a single passage (Figure 4E). Moreover, following two passages, the WNV-NY/GLuc(ALIC➔APC)-pp2 population consisted almost entirely of virus lacking a substantial portion of the GLuc(ALIC➔APC) insert. Since the PCR products were distinctly larger than that of wild-type WNV-NY, the p2 population most likely consisted of virus retaining only a portion of the GLuc(ALIC➔APC) insert. Sequence analysis of the WNV-NY/GLuc(ALIC➔APC)-pp2 p2 PCR product returned a mixed sequence, indicative of the presence of multiple virus species. Thus, the GLuc insertion is not well tolerated at the C/CA junction. Furthermore, the instability of the GLuc(ALIC➔APC) mutant virus (Figure 4B–E) suggests that the ALIC➔APC mutation does not provide any appreciable fitness advantage, though WNV-NY/GLuc(ALIC➔APC)-pp1 may contain compensatory mutations outside of the C/CA region that offer some stability to the recombinant virus. The inability to recover a WNV-NY/GLuc virus containing an insertion of comparable size to TAT(1-25) (~75 nts) suggests that, in addition to size, the sequence of any additional nucleotides at the C/CA junction influences the stability of resulting viruses. Therefore, this site may tolerate an insertion larger than 75 nts if the sequence is sufficiently inert.

Numerous GLuc-reactive bands were detected in WNV-NY/GLuc(ALIC➔APC)-pp1 or -pp2-infected cells [26], indicating that the GLuc reporter was translated. However, we did not detect GLuc activity in cell lysates or culture supernatants of Vero cells infected with WNV-NY/GLuc(ALIC➔APC)-pp1 or -pp2 (Figure S3), suggesting that the virus does not produce functional GLuc. The lack of functional GLuc may be due to the ALIC➔APC mutation altering the protein’s activity. Alternatively, within the context of the virus, GLuc may not be properly folded, or incomplete cleavage of GLuc from the rest of the WNV polyprotein may impede GLuc function. 

### 2.4. Effect of Insertions on WNV Replication

**Figure 5:**
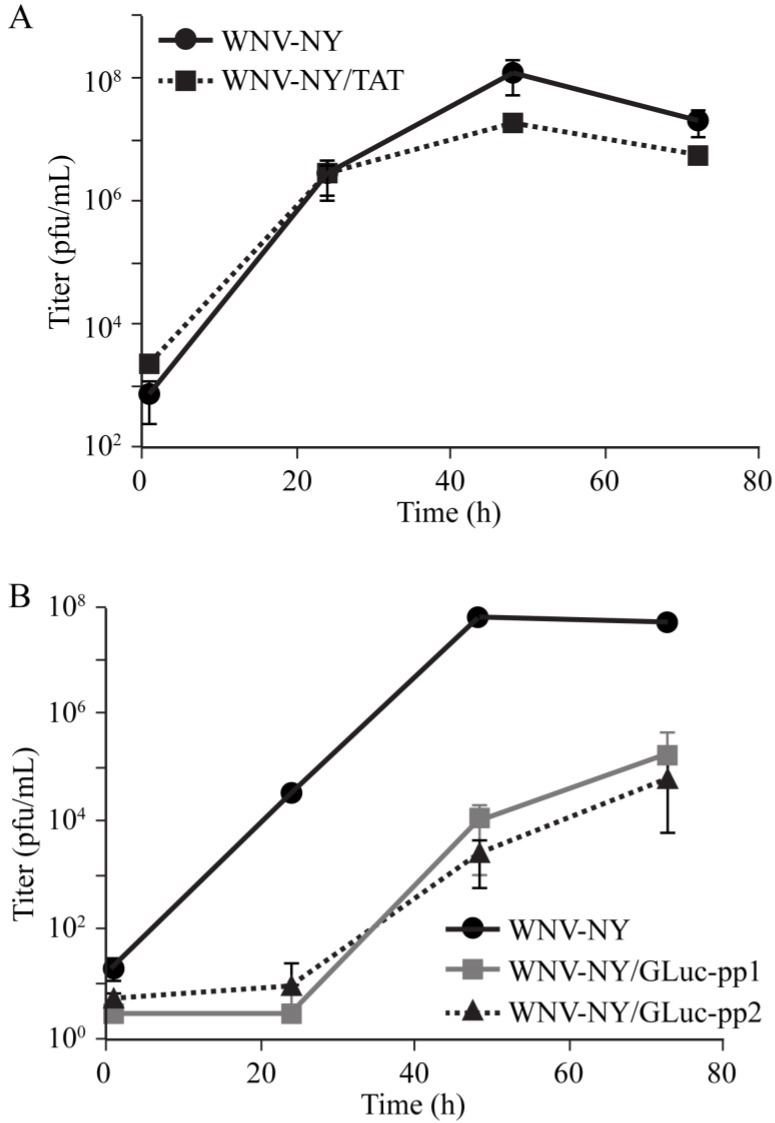
Replication of WNV-NY recombinant viruses. (**A**) WNV-NY/TAT(1-25). Vero monolayers were infected (MOI = 0.05) with WNV-NY or WNV-NY/TAT(1-25) p0; (**B**) WNV-NY/GLuc(ALIC➔APC). Vero monolayers were infected (MOI = 0.005) with WNV-NY or WNV-NY-GLuc(ALIC➔APC)-pp1 or -pp2 p0. Culture supernatants were collected at the indicated times after infection and infectious particle production determined by plaque assay on Vero cells. Values represent the average number of pfu per mL of supernatant (± standard deviation) from three independent experiments.

Because the truncated WNV-NY/TAT(1-25) was stable, we hypothesized that the TAT(1-25) insertion had little effect on virus fitness. Therefore, we compared wild-type WNV-NY and WNV-NY/TAT(1-25) replication in Vero cells. Both viruses exhibited similar replication kinetics and reached peak titers at 48 h post infection (Figure 5A). While we did observe slightly higher peak viral titers for wild-type WNV-NY compared to WNV-NY/TAT(1-25), the difference was not statistically significant. Thus, insertion of the TAT(1-25) sequence did not significantly alter WNV-NY fitness.

Unlike the TAT(1-25) insert, the GLuc inserts were unstable in WNV-NY, suggesting that they conferred a fitness disadvantage to the virus. To test this hypothesis, we also compared the replication kinetics of WNV-GLuc(ALIC➔APC)-pp1 and -pp2 with wild-type WNV-NY. WNV-NY replication was readily detected by 24 h after infection and reached peak levels by 48 h (Figure 5B). In comparison, WNV-NY/GLuc(ALIC➔APC)-pp1 and -pp2 infectious particle production was delayed and peak virus levels, which occurred at 72 h, were significantly reduced. Thus, unlike WNV-NY/TAT(1-25), WNV-NY/GLuc(ALIC➔APC) had a substantial growth disadvantage compared to wild-type virus, indicating that the GLuc(ALIC➔APC) insert reduced viral fitness.

## 3. Experimental Section

### 3.1. Cells

Vero cells were maintained at 37 °C and 5% CO_2_ in Dulbecco’s modified Eagle medium (DMEM) (Mediatech) supplemented with 10% fetal bovine serum (FBS) (BioWhittaker), nonessential amino acids, 4.5 mg/L glucose, L-glutamine, and sodium pyruvate, 10 U/mL penicillin, 10 μg/mL streptomycin, and 25 ng/mL amphotericin B (complete DMEM). Mosquito C6/36 cells were maintained at 28 °C and 5% CO_2_ in Minimum essential medium (MEM) (Mediatech) supplemented as described for DMEM. 

### 3.2. Plasmid Construction

**Figure 6:**
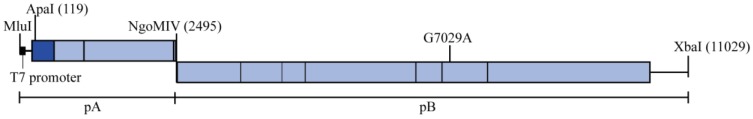
Construction of full-length WNV infectious clones. The WNV genome was separated onto two plasmids: pA and pB. pA contained the WNV 5’ UTR, WNV structural genes, and the inserted reporter gene (see Figure 1B), under control of the T7 promoter. The location of the ApaI site used for cloning the pA construct is shown. pB contained the WNV non-structural genes and 3’ UTR, immediately proceeded by an XbaI site. The location of the G7029A mutation removing the NgoMIV site is indicated. All numbers are the nt positions based on the WNV-NY strain 3356 (Accession number AF404756) sequence, excluding any additional nts added due to reporter insertion.

Plasmids TX-AB and TX-CG [29] (a kind gift from Michael Gale Jr.) were used to generate the pA-NY and pB-NY plasmids, respectively (Figure 6). Plasmid pA-NY was generated by replacing the ApaI/NgoMIV fragment (WNV nts 119-2495) of TX-AB with the corresponding fragment from pFLWNV [30]. To generate pB-NY, pFLWNV was used as template for PCR to amplify WNV-NY fragments 2494-7037 and 7024-11029 using primers encoding a G➔A point mutation at position 7029, which removed the NgoMIV restriction site at position 7027 in both fragments. The resulting PCR fragments were assembled with the vector backbone of TX-CG (NgoMIV and XbaI digested) using Gibson Assembly Mastermix (New England Biolabs, Ipswich, MA, USA), as per the manufacturer’s directions.

An HIV-TAT-encoding cassette consisting of WNV-NY nts 290-1094 was synthesized by GenScript (Piscataway, NJ, USA) and inserted into a pUC57 vector. The synthesized cassette contained the coding region for HIV-TAT(1-67), flanked by 5’ GGAGGA (GlyGly) and 3’ AAGAGG (LysArg) sequences, inserted between nts 414 and 415 (C/CA junction) of WNV-NY strain 3356. The pUC57-WNV-NY/TAT plasmid was used as template for PCR amplification to generate a DNA fragment corresponding to the synthesized cassette. A similar cassette containing the Gaussia luciferase (GLuc) gene [27,28] in place of the TAT(1-67) sequence was synthesized as three overlapping gBlocks spanning WNV-NY nts 265-1122 (IDT). The WNV-NY/TAT PCR product or the overlapping GLuc gBlocks were inserted into ClaI-digested pA-NY using Gibson Assembly Mastermix. Sequence differences between the generated pA and pB plasmids and WNV strain 3356 (wild-type) are indicated in Table 1. 

**Table 1:** Sequence differences between the infectious clone pA and pB plasmids and WNV strain 3356.

Position ^1^	NY-3356 Genome	IC Plasmids	Amino Acid Change	Location
1285	T	C	Silent	E
3840	T	C	Silent	NS2A
7029^2^	G	A	Silent	NS4B
7826	T	C	V➔A	NS5
8067	G	A	Silent	NS5
9123	C	T	Silent	NS5
10613	C	T	Silent	3’ UTR
10783	C	T	Silent	3’ UTR

^1^ Nucleotide position and sequence based on WNV-NY strain 3356 (Accession number AF404756); ^2^ Mutation engineered to remove NgoMIV endonuclease site, facilitating digestion/ligation of pA and pB plasmids (Figure 6).

### 3.3. RNA Transcription and Transfection

Full-length linear DNA templates for *in vitro* transcription were prepared by ligating pA-NY (MluI and NgoMIV digested) with pB-NY (NgoMIV and XbaI digested). Briefly, purified pA and pB products were combined at a 1:1 molar ratio, precipitated, resuspended in water, and ligated overnight. The ligated DNA was treated with 0.8 μg Proteinase K for 1 h at 37 °C, purified by phenol: chloroform extraction, precipitated, and resuspended in 4–6 μL of RNase-free water. The purified WNV-NY/TAT or WNV-NY/GLuc ligation reactions (1 μg) served as templates for *in vitro* transcription using the AmpliCap-Max^TM^ T7 High Yield Message Maker Kit (Cell Script). *In vitro*-transcribed RNA was purified by phenol: chloroform extraction, precipitated with 5M ammonium acetate, and resuspended in water. For transfection into Vero cells, 12 μg of RNA was transfected into 1 × 10^6^ cells using the Neon^®^ Transfection system (Invitrogen, Carlsbad, CA, USA) with the following settings: 1150 V, 20 ms, and 2 pulses. Unless otherwise stated, culture supernatants were collected 7 days after transfection and clarified by centrifugation at 1500 × *g* for 5 min. 

### 3.4. Wild-Type WNV-NY

Working stocks of WNV-NY strain 3356 (Accession number AF404756) were generated from the infectious clone pFLWNV [30]. Briefly, infectious particles were recovered as previously described [30], passaged once in 293 cells at a low multiplicity of infection (MOI), and subsequently passaged in Vero cells. The titer of the viral stock was determined by plaque assay (see below) on Vero cells. 

### 3.5. Plaque Assays

Monolayers of Vero cells in six-well plates were washed with Dulbecco’s phosphate buffered saline (DPBS) (Hyclone) followed by the addition of serial dilutions of viral samples. The cells were incubated at 37 °C/5% CO_2_ for 1 h with rocking, the inoculums removed, and a 0.9% agarose-complete DMEM overlay added. Cell monolayers were incubated for 48 h and a second overlay of agarose-complete DMEM containing 0.003% neutral red (MP Biomedicals) was added. Plaques were counted 4 days after initial inoculation. 

### 3.6. Amplification of Recombinant Viruses

Vero cells or C3/36 monolayers in a T75 flask or 10-cm dish, respectively, were washed once with DPBS and inoculated with 1 mL of virus stock. Cultures were incubated for 1 h in 5% CO_2_ at the appropriate temperature, after which 9 mL of complete DMEM or MEM was added to the cells. Culture supernatants were collected at 48 h post-infection and clarified by centrifugation at 1500 × *g* for 5 min. For serial passage experiments, 1 mL (WNV-NY/TAT) p1 stocks or 500 μL of p1 stocks diluted 1:2 in DMEM (WNV-NY/GLuc) was used for inoculations to generate p2 virus. For all subsequent passages, cells were inoculated with 50 μL of virus and diluted 1:20 in DMEM (total volume = 1 mL).

### 3.7. Reverse Transcription-PCR Analysis

Total RNA was extracted from monolayers using TriZOL Reagent (Invitrogen), as per the manufacturer’s directions. The extracted RNA was used as template for reverse transcription with M-MLV (New England Biolabs, Ipswich, MA, USA) using a WNV-specific antisense primer (WNV-500as: 5’ ATC ATC ACC TTC CCT TGG AAG TTA 3’), which anneals at the end of the CA sequence in the WNV genome. The resulting cDNA was used as template for PCR with the WNV-500as and WNV-29s (sense) (5’ AGT GAT ATC GAT GTC TAA GAA ACC AGG AGG GCC C 3’) primers (Figure 1B). PCR products were analyzed by agarose-gel electrophoresis (2% agarose) followed by staining with ethidium bromide. For sequencing, reactions with a single product were purified directly using the Wizard SV Gel and PCR clean up kit (Promega). When more than one major PCR product was detected, the bands of interest were gel purified. Purified products were sent to GeneWiz for sequencing using WNV-500as and the resulting sequences analyzed using MacVector. 

### 3.8. Growth Curves

Vero cell monolayers were washed once with DPBS and infected with WNV at an MOI of 0.05 or 0.005. After 1 h at 37 °C/5% CO_2_, the inoculum was replaced with complete DMEM. Culture supernatants were collected at the indicated times after initial infection and clarified by centrifugation at 1500 × *g* for 5 min. 

### 3.9. Immunoblot Analyses

Cell monolayers were washed with DPBS, lysed with RIPA buffer (10 mM Tris pH 7.4, 150 mM NaCl, 0.02% NaN_3_, 1% sodium deoxycholate, 1% Triton X-100, 0.1% sodium dodecyl sulfate [SDS], and 1X protease inhibitors [Sigma]), and subjected to SDS-polyacrylamide gel electrophoresis (PAGE). Proteins were transferred to nitrocellulose membranes that were then incubated with the following antiserum: monoclonal mouse α-HIV TAT (1:3000) (Abcam; ab42359; detects residues 2-9 of HIV-TAT), polyclonal mouse α-WNV (1:1000) (Arbovirus Resource Center; T35570; detects all WNV proteins), and monoclonal mouse α-Actin (1:400) (Abcam; ab3280).

### 3.10. Plaque Purification

Individual plaques were picked using a sterile filter P1000 tip (VWR) and placed in 1 mL of complete DMEM. After 4 h at 4 °C, the plaque-containing media was used to inoculate Vero monolayers in T75 flasks. Following 1 h of rocking at 37 °C/5% CO_2_, 9 mL of complete DMEM was added to each flask. Culture supernatants were collected at 96 h post-infection and clarified by centrifugation at 1500 × *g* for 5 min.

## 4. Conclusions

High throughput screening of compounds with potential anti-WNV activity has been hindered by the lack of a WN reporter virus with wild-type replication. In this study, we generated two recombinant WNVs. As with previously generated WN reporter viruses [14,15], both WNV-NY/TAT and WNV-NY/GLuc were unstable and exhibited decreased fitness compared to wild-type WNV-NY. While the previously generated WN reporter viruses targeted the 3’ UTR as the site of reporter insertion, we inserted coding sequence at the C/CA junction. The instability of the resulting viruses, therefore, indicates that not only the 3’ UTR, but also the 5’ coding region of WNV is refractory towards the insertion of large coding sequences. However, nts 1-75 of HIV-TAT were stably integrated into WNV at this site. Moreover, insertion of these nucleotides did not significantly alter virus replication. Thus, the C/CA junction is amenable to genetic manipulation. The function and sequence of TAT(1-67) and GLuc vary greatly, suggesting that size is the primary factor restricting nucleotide insertions at the C/CA junction. Whether other junctions within the polyprotein are more flexible in regards to the insertion of large sequences is unclear, though the instability of the other WN reporter viruses suggests that size constraints on insertions may exist regardless of their placement within the viral genome. Nevertheless, as technologies advance, the discovery and design of smaller reporters may facilitate the generation of a recombinant WNV containing a stable reporter.

## Supplementary Files

Supplementary File 1 Supplementary Figures (PDF, 361 KB)

## References

[1] Murray K.O., Mertens E., Despres P. (2010). West Nile virus and its emergence in the United States of America. Vet. Res..

[2] Sejvar J.J., Marfin A.A. (2006). Manifestations of West Nile neuroinvasive disease. Rev. Med. Virol..

[3] Hayes C.G. (2001). West Nile virus: Uganda, 1937, to New York City, 1999. Ann. N. Y. Acad. Sci..

[4] Petersen L.R., Roehrig J.T. (2001). West Nile virus: a reemerging global pathogen. Emerg. Infect. Dis..

[5] Savage H.M., Ceianu C., Nicolescu G., Karabatsos N., Lanciotti R., Vladimirescu A., Laiv L., Ungureanu A., Romanca C., Tsai T.F. (1999). Entomologic and avian investigations of an epidemic of West Nile fever in Romania in 1996, with serologic and molecular characterization of a virus isolate from mosquitoes. A. J. Trop. Med. Hyg..

[6] CDC. http://www.cdc.gov/westnile/index.html/.

[7] Moritoh K., Maeda A., Nishino T., Sasaki N., Agui T. (2011). Development and application of West Nile virus subgenomic replicon RNA expressing secreted alkaline phosphatase. J. Vet. Med. Sci..

[8] Shan C., Li X., Deng C., Shang B., Xu L., Ye H., Yuan Z., Zhang B. (2013). Development and characterization of West Nile virus replicon expressing secreted Gaussia luciferase. Virol. Sin..

[9] Alcaraz-Estrada S.L., Reichert E.D., Padmanabhan R. (2013). Construction of self-replicating subgenomic West Nile virus replicons for screening antiviral compounds. Methods Mol. Biol..

[10] Gu B., Ouzunov S., Wang L., Mason P., Bourne N., Cuconati A., Block T.M. (2006). Discovery of small molecule inhibitors of West Nile virus using a high-throughput sub-genomic replicon screen. Antivir. Res..

[11] Noueiry A.O., Olivo P.D., Slomczynska U., Zhou Y., Buscher B., Geiss B., Engle M., Roth R.M., Chung K.M., Samuel M. (2007). Identification of novel small-molecule inhibitors of West Nile virus infection. J. Virol..

[12] Puig-Basagoiti F., Deas T.S., Ren P., Tilgner M., Ferguson D.M., Shi P.Y. (2005). High-throughput assays using a luciferase-expressing replicon, virus-like particles, and full-length virus for West Nile virus drug discovery. Antimicrob. Agents Chemother..

[13] Puig-Basagoiti F., Qing M., Dong H., Zhang B., Zou G., Yuan Z., Shi P.Y. (2009). Identification and characterization of inhibitors of West Nile virus. Antivir. Res..

[14] Pierson T.C., Diamond M.S., Ahmed A.A., Valentine L.E., Davis C.W., Samuel M.A., Hanna S.L., Puffer B.A., Doms R.W. (2005). An infectious West Nile virus that expresses a GFP reporter gene. Virology.

[15] Deas T.S., Binduga-Gajewska I., Tilgner M., Ren P., Stein D.A., Moulton H.M., Iversen P.L., Kauffman E.B., Kramer L.D., Shi P.Y. (2005). Inhibition of flavivirus infections by antisense oligomers specifically suppressing viral translation and RNA replication. J. Virol..

[16] Shi P.Y., Tilgner M., Lo M.K. (2002). Construction and characterization of subgenomic replicons of New York strain of West Nile virus. Virology.

[17] McGee C.E., Shustov A.V., Tsetsarkin K., Frolov I.V., Mason P.W., Vanlandingham D.L., Higgs S. (2010). Infection, dissemination, and transmission of a West Nile virus green fluorescent protein infectious clone by Culex pipiens quinquefasciatus mosquitoes. Vector Borne Zoonotic Dis..

[18] Schrauf S., Mandl C.W., Bell-Sakyi L., Skern T. (2009). Extension of flavivirus protein C differentially affects early RNA synthesis and growth in mammalian and arthropod host cells. J. Virol..

[19] Nowak T., Farber P.M., Wengler G., Wengler G. (1989). Analyses of the terminal sequences of West Nile virus structural proteins and of the *in vitro* translation of these proteins allow the proposal of a complete scheme of the proteolytic cleavages involved in their synthesis. Virology.

[20] Yamshchikov V.F., Compans R.W. (1993). Regulation of the late events in flavivirus protein processing and maturation. Virology.

[21] Yamshchikov V.F., Compans R.W. (1994). Processing of the intracellular form of the West Nile virus capsid protein by the viral NS2B-NS3 protease: An *in vitro* study. J. Virol..

[22] Kuppuswamy M., Subramanian T., Srinivasan A., Chinnadurai G. (1989). Multiple functional domains of Tat, the trans-activator of HIV-1, defined by mutational analysis. Nucl. Acids Res..

[23] Nall T.A., Chappell K.J., Stoermer M.J., Fang N.X., Tyndall J.D., Young P.R., Fairlie D.P. (2004). Enzymatic characterization and homology model of a catalytically active recombinant West Nile virus NS3 protease. J. Biol. Chem..

[24] Shiryaev S.A., Kozlov I.A., Ratnikov B.I., Smith J.W., Lebl M., Strongin A.Y. (2007). Cleavage preference distinguishes the two-component NS2B-NS3 serine proteinases of Dengue and West Nile viruses. Biochem. J..

[25] Shiryaev S.A., Ratnikov B.I., Chekanov A.V., Sikora S., Rozanov D.V., Godzik A., Wang J., Smith J.W., Huang Z., Lindberg I. (2006). Cleavage targets and the D-arginine-based inhibitors of the West Nile virus NS3 processing proteinase. Biochem. J..

[26] Vandergaast R., Fredericksen B.L. (2013).

[27] Tannous B.A., Kim D.E., Fernandez J.L., Weissleder R., Breakefield X.O. (2005). Codon-optimized Gaussia luciferase cDNA for mammalian gene expression in culture and *in vivo*. Mol. Ther..

[28] Verhaegent M., Christopoulos T.K. (2002). Recombinant Gaussia luciferase. Overexpression, purification, and analytical application of a bioluminescent reporter for DNA hybridization. Anal. Chem..

[29] Suthar M.S., Brassil M.M., Blahnik G., Gale M. (2012). Infectious clones of novel lineage 1 and lineage 2 West Nile virus strains WNV-TX02 and WNV-Madagascar. J. Virol..

[30] Shi P.Y., Tilgner M., Lo M.K., Kent K.A., Bernard K.A. (2002). Infectious cDNA clone of the epidemic West Nile virus from New York City. J. Virol..

