# Supplementary material for: Generation of West Nile Virus Infectious Clones Containing Amino Acid Insertions Between Capsid and Capsid Anchor
Source: Viruses. 2014 Apr 9;6(4):1637–53. doi: 10.3390/v6041637 (PMC4014714; doi:10.3390/v6041637)

Figure S1. TAT(1-67) containing additional amino acids at the C- and N-terminal ends is a functional trans-activator. TAT(1-67) and GlyGly-TAT(1-67)-LysArg (GG-TAT(1-67)-KR) eukaryotic expression constructs were generated by inserting the sequences encoding TAT(1-67) and GG-TAT(1-67)-KR into the multiple cloning site of pEFBOS. To assess the functionality of GG-TAT(1-67)-KR, 1 μg of pEFBOS-TAT(1-67), pEFBOS-GG-TAT(1-67)-KR, or empty pEFBOS vector was transfected into TZM-bl cells, which are stably transfected with an HIV LTR-driven firefly luciferase construct, using Lipofectamine2000. Cell-lysates prepared 44 h after transfection were subjected to SDS-PAGE and immunoblot analysis with anti-Luciferase antibodies. Protein molecular size standards (in kilodaltons) are indicated on the left.

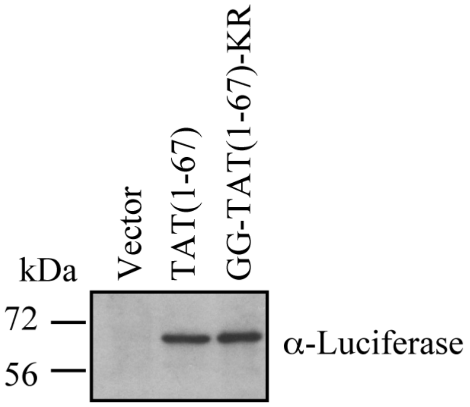

Figure S2. WNV-NY/TAT(1-25) does not produce functional TAT. TZM-bl monolayers were infected (MOI = 1) with WNV-NY or WNV-NY/TAT(1-25) p1 or were mock-infected. After 64 h, cells were lysed in 1X Passive Lysis Buffer (Promega) containing 1% Triton X-100. Firefly luciferase activity in the lysates was measured using the Luciferase Assay Reagent II component of a Dual-Luciferase® Reporter Assay System (Promega), according to the manufacturer's directions. Signal was quantified on a Berthold Centro XS3 LB960 luminometer. Values represent the average (± standard deviation) firefly luciferase activity in relative light units (RLU) from triplicate samples.

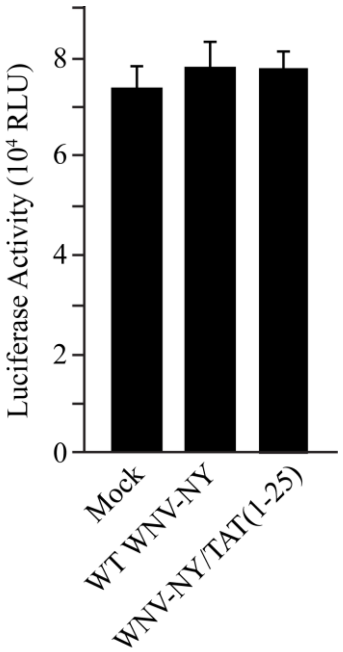

Figure S3. WNV-NY/GLuc(ALIC→APC)-pp1 and pp2 do not produce functional GLuc. Vero monolayers were infected (MOI = 0.01) with WNV-NY, WNV-NY/GLuc(ALIC→APC)-pp1, or WNV-NY/GLuc(ALIC→APC)-pp2 or were mock infected. After 68 h, culture supernatants were collected and virus was inactivated by adding Triton X-100 to a final concentration of 1%. The Vero monolayers were washed and lysed in 1X Passive Lysis Buffer (Promega) containing 1% Triton X-100. Gaussia Luciferase activity was measured using a BioLux Gaussia Luciferase Assay Kit (NEB) and quantified on a Berthold Centro XS3 LB960 luminometer. Secreted signal was determined from the inactivated supernatants, while cellular signal was calculated from the cell lysates. As a positive control, samples were prepared from Vero cells transfected 68 h earlier with a eukaryotic GLuc expression construct. Values represent the average (± standard deviation) relative light units (RLU) from two independent experiments.

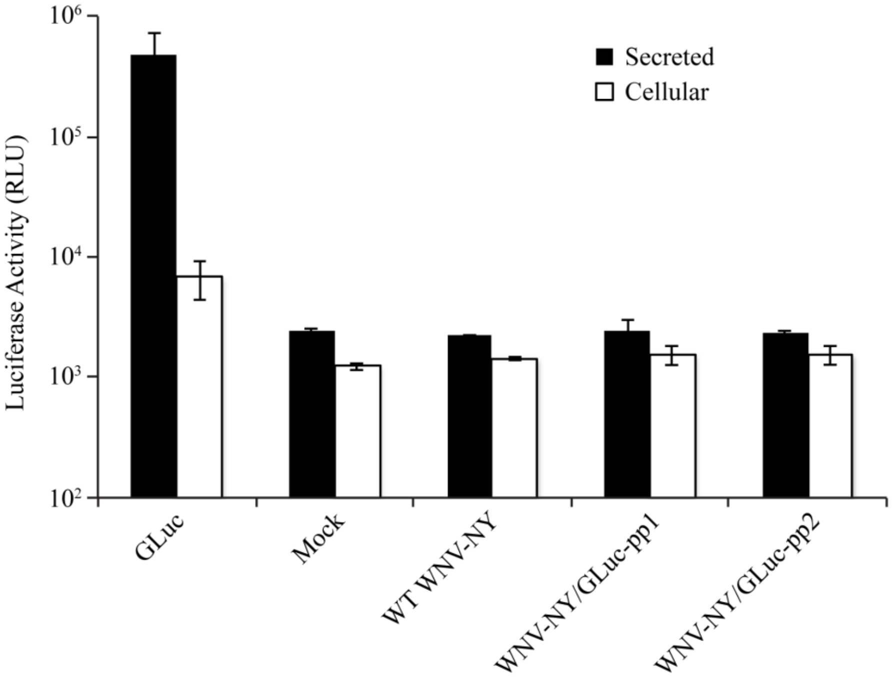

Supplement: Supplementary File 1 — Supplementary Figures (PDF, 361 KB) [file viruses-06-01637-s001.pdf]
